# Supplementary figures and images for: Distinct mechanisms control the specific synaptic functions of Neuroligin 1 and Neuroligin 2
Source: EMBO Rep. 2025 Jan 2;26(3):860–79. doi: 10.1038/s44319-024-00286-4 (PMC11811269; doi:10.1038/s44319-024-00286-4)

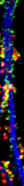

Supplement: Supplementary file 2 — Source data Fig. 1 [file 44319_2024_286_MOESM2_ESM.zip › Fig1/Fig1C/zoom-Cre+NL1-WT.tif]

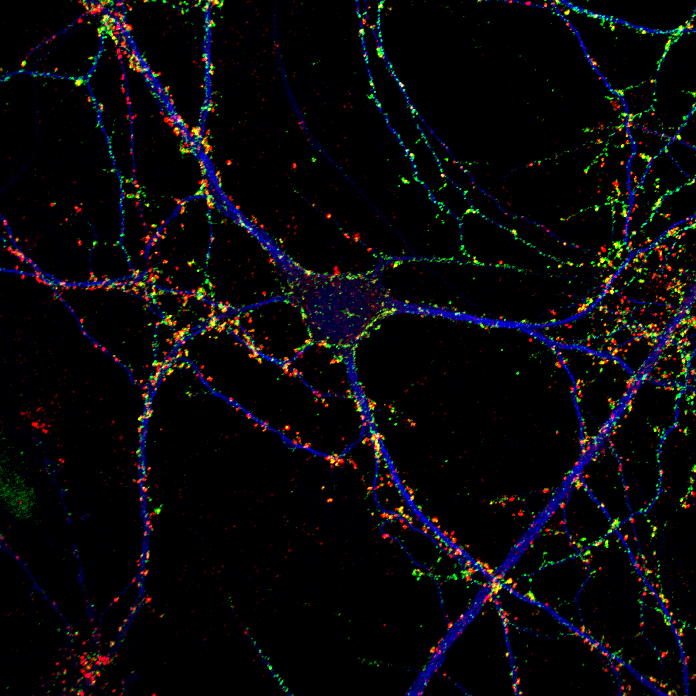

Supplement: Supplementary file 2 — Source data Fig. 1 [file 44319_2024_286_MOESM2_ESM.zip › Fig1/Fig1C/Merge-Cre+NL1-WT.tif]

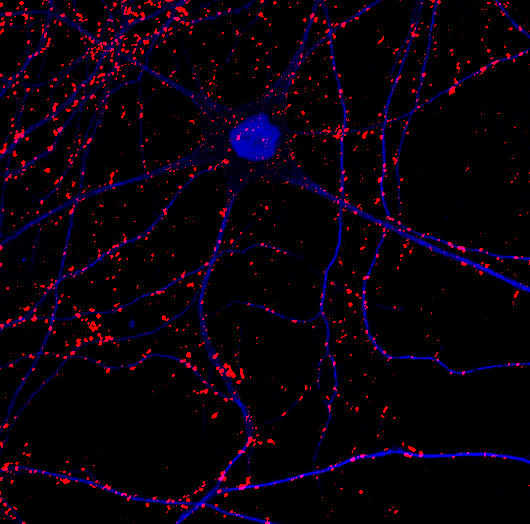

Supplement: Supplementary file 2 — Source data Fig. 1 [file 44319_2024_286_MOESM2_ESM.zip › Fig1/Fig1C/MAP2+Homer1-Cre+NL2WT.tif]

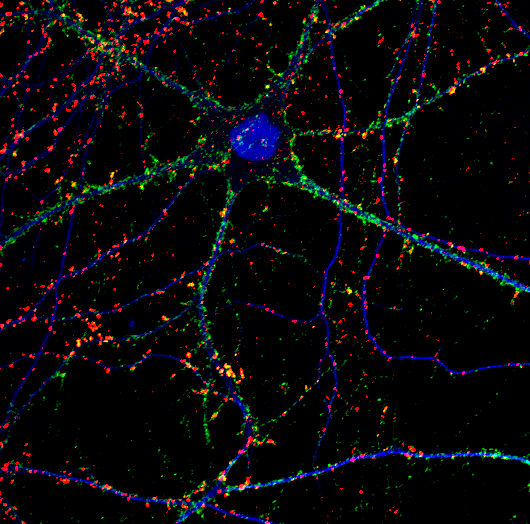

Supplement: Supplementary file 2 — Source data Fig. 1 [file 44319_2024_286_MOESM2_ESM.zip › Fig1/Fig1C/Merge-Cre+NL2WT.tif]

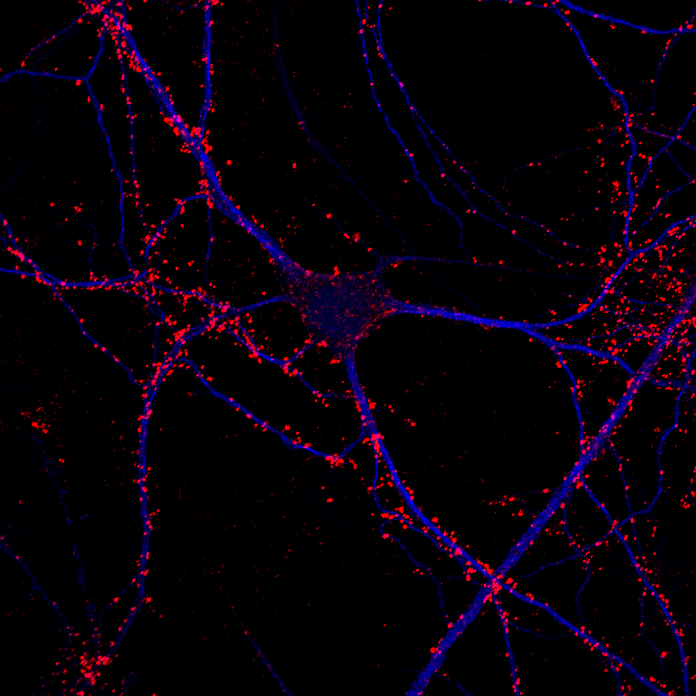

Supplement: Supplementary file 2 — Source data Fig. 1 [file 44319_2024_286_MOESM2_ESM.zip › Fig1/Fig1C/MAP2+Homer1-Cre+NL1-WT.tif]

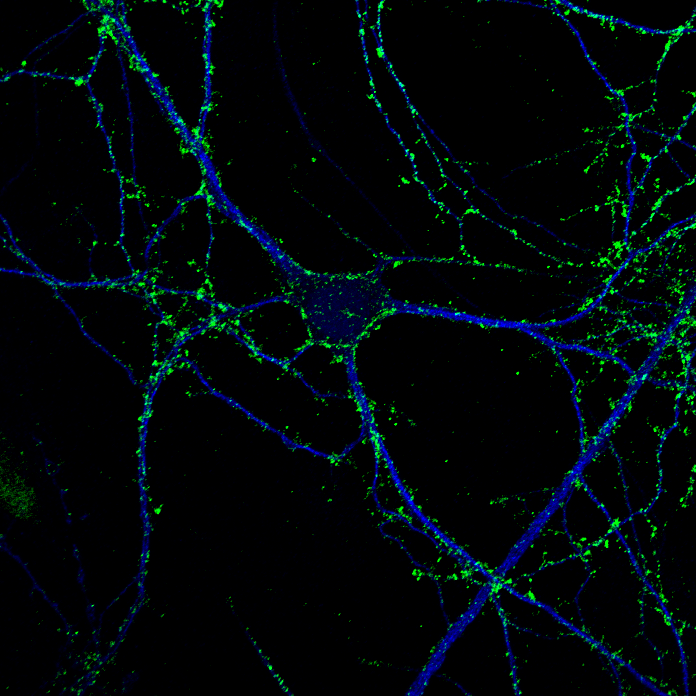

Supplement: Supplementary file 2 — Source data Fig. 1 [file 44319_2024_286_MOESM2_ESM.zip › Fig1/Fig1C/MAP+HA-Cre+NL1-WT.tif]

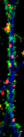

Supplement: Supplementary file 2 — Source data Fig. 1 [file 44319_2024_286_MOESM2_ESM.zip › Fig1/Fig1C/zoom-Cre+NL2WT.tif]

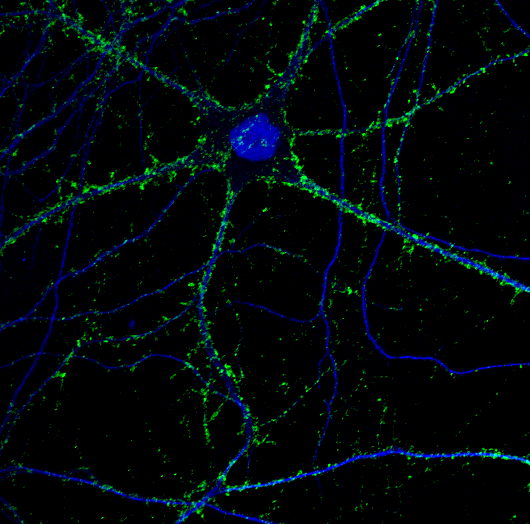

Supplement: Supplementary file 2 — Source data Fig. 1 [file 44319_2024_286_MOESM2_ESM.zip › Fig1/Fig1C/MAP2+HA- Cre+NL-2WT.tif]

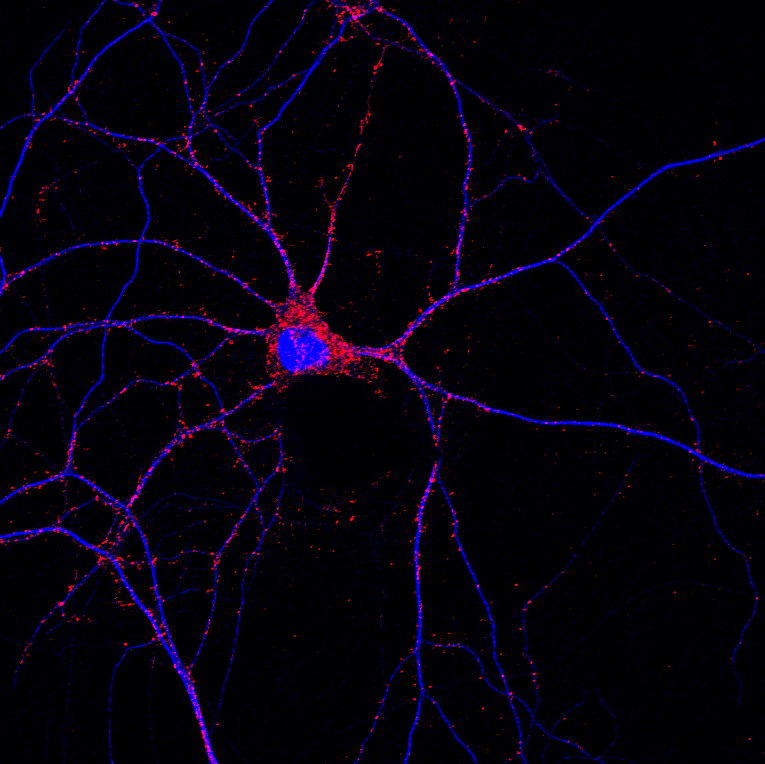

Supplement: Supplementary file 2 — Source data Fig. 1 [file 44319_2024_286_MOESM2_ESM.zip › Fig1/Fig1E/NL1-WT-MAP2+gephyrin.tif]

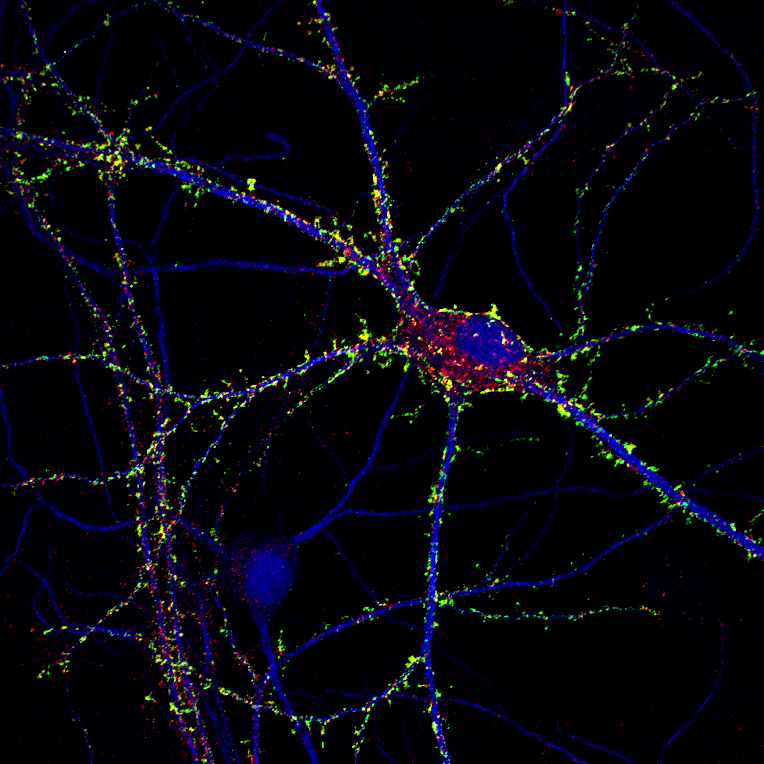

Supplement: Supplementary file 2 — Source data Fig. 1 [file 44319_2024_286_MOESM2_ESM.zip › Fig1/Fig1E/NL2-WT-merge.tif]

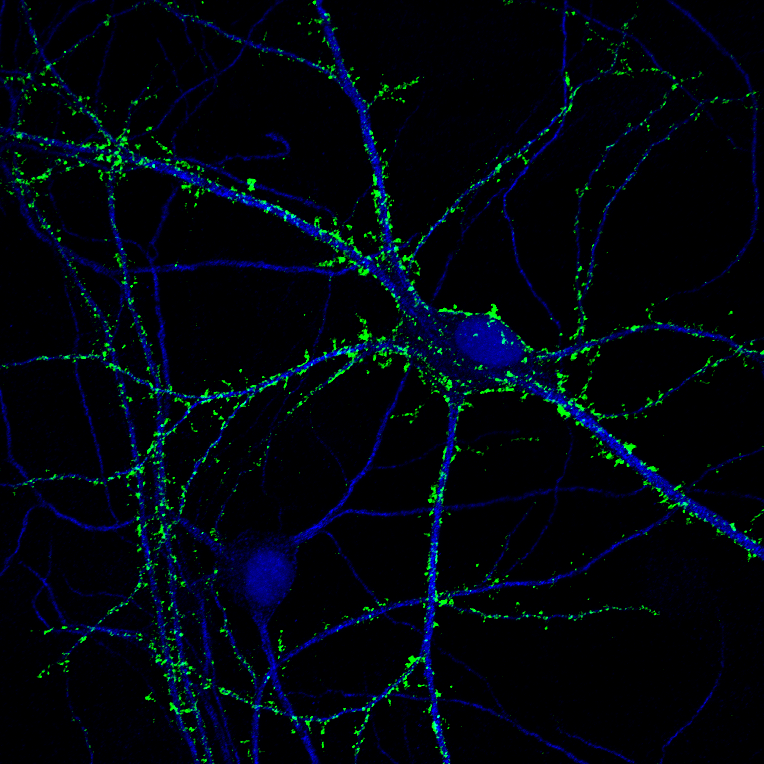

Supplement: Supplementary file 2 — Source data Fig. 1 [file 44319_2024_286_MOESM2_ESM.zip › Fig1/Fig1E/NL2-WT-MAP2+HA.tif]

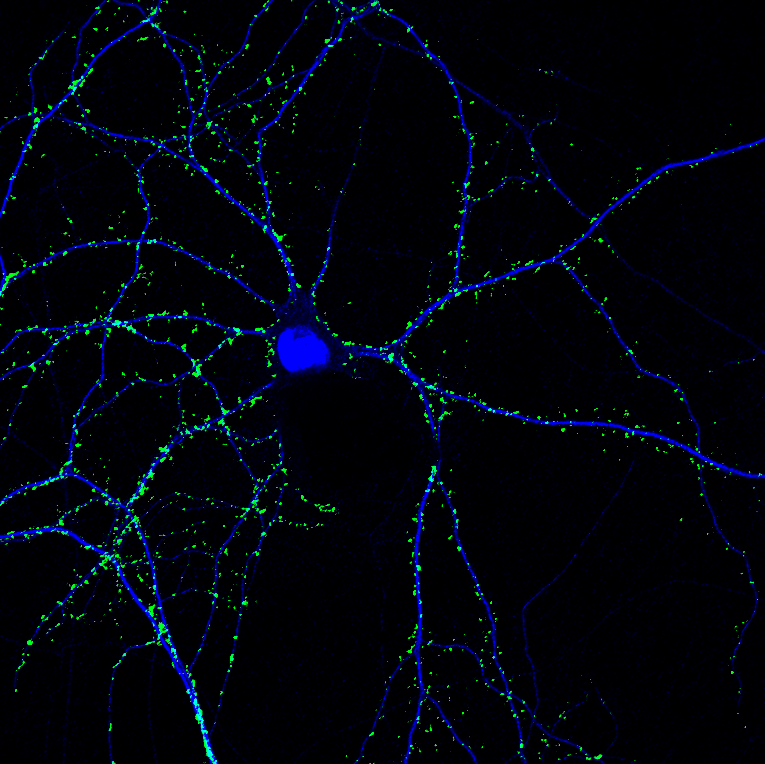

Supplement: Supplementary file 2 — Source data Fig. 1 [file 44319_2024_286_MOESM2_ESM.zip › Fig1/Fig1E/NL1-WT-MAP2+HA.tif]

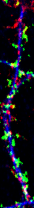

Supplement: Supplementary file 2 — Source data Fig. 1 [file 44319_2024_286_MOESM2_ESM.zip › Fig1/Fig1E/NL1-WT-zoom.tif]

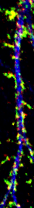

Supplement: Supplementary file 2 — Source data Fig. 1 [file 44319_2024_286_MOESM2_ESM.zip › Fig1/Fig1E/NL2-WT-zoom..tif]

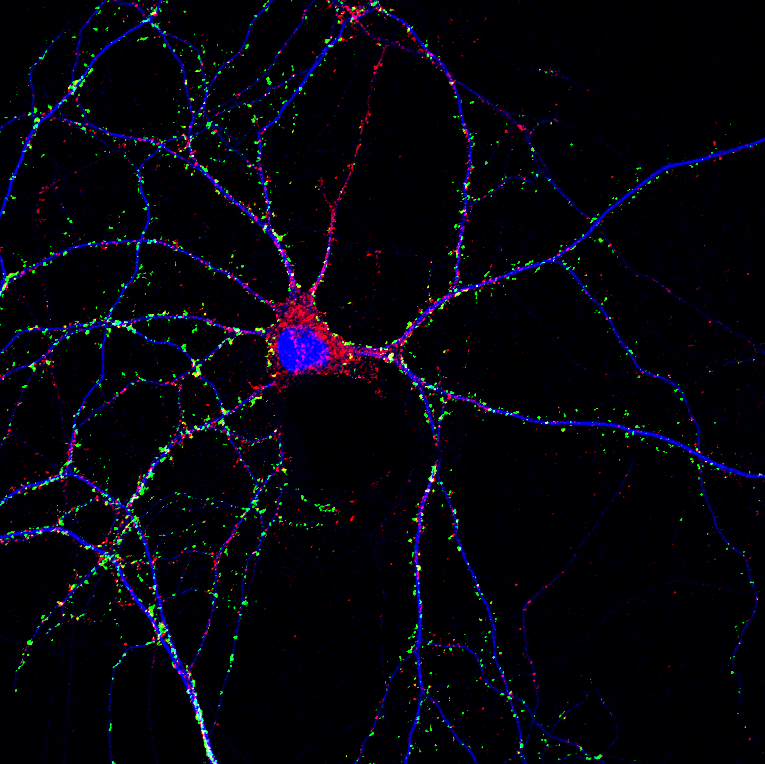

Supplement: Supplementary file 2 — Source data Fig. 1 [file 44319_2024_286_MOESM2_ESM.zip › Fig1/Fig1E/NL1-WT-merge.tif]

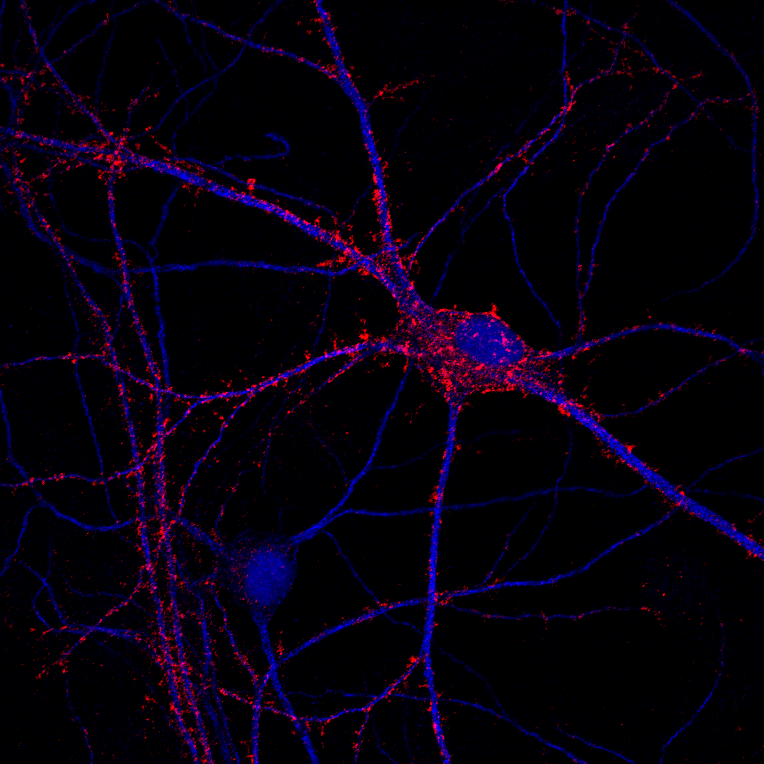

Supplement: Supplementary file 2 — Source data Fig. 1 [file 44319_2024_286_MOESM2_ESM.zip › Fig1/Fig1E/NL2-WT-MAP2+gephyrin.tif]
