# Supplementary material for: Distinct mechanisms control the specific synaptic functions of Neuroligin 1 and Neuroligin 2
Source: EMBO Rep. 2025 Jan 2;26(3):860–79. doi: 10.1038/s44319-024-00286-4 (PMC11811269; doi:10.1038/s44319-024-00286-4)
Supplement: Supplementary file 10 — Expanded View Figures [file 44319_2024_286_MOESM10_ESM.pdf]

# Expanded View Figures

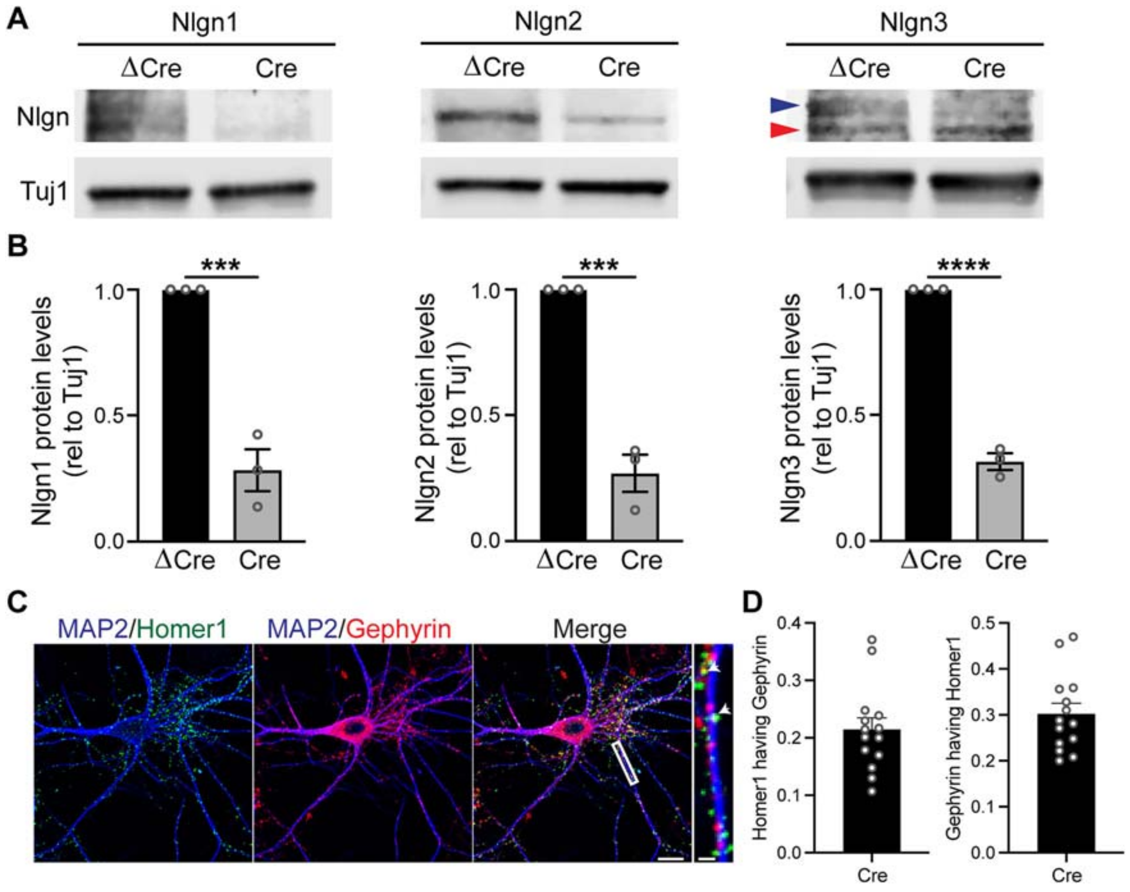


**Figure EV1. Neuroligin protein measurement and co-staining Homer1 with Gephyrin in Nlgn1234 cKO cultured hippocampal neurons (related to Fig. 1).**

(**A**) Representative images of western blot for Nlgn1, Nlgn2, and Nlgn3 protein expressions from DIV14-16 cultured Nlgn1234 conditional knockout mice neurons, infected with either ΔCre or Cre. Note that the Nlgn3 antibody detects Nlgn3 (blue arrow) and nonspecific band (red arrow). (**B**) Summary graphs of western blot analysis for Nlgn1, Nlgn2, and Nlgn3 protein expressions. (Bar and line graphs indicate mean ± SEM; samples/experiments = 3/3. 3 technical replicates. Statistical significance was assessed by unpaired t test, ****p* < 0.001; *****p* < 0.0001). (**C**) Representative image from DIV14-16 cultured Nlgn1234 conditional knockout mice neurons. The neuron was labeled with antibodies to Homer1 (Green), Gephyrin (red), and MAP2 (blue). Scale bar: 20 µm. The right panels show an enlarged box area (arrowheads indicate Homer1 puncta overlapped with Gephyrin puncta) Scale bar: 5 µm. (**D**) Summary graph of Homer1 overlap percentage with Gephyrin and Gephyrin overlap percentage with Homer1 (Bar and line graphs indicate mean ± SEM; numbers of cells/experiment = 14/1). Source data are available online for this figure.

 © The Author(s)

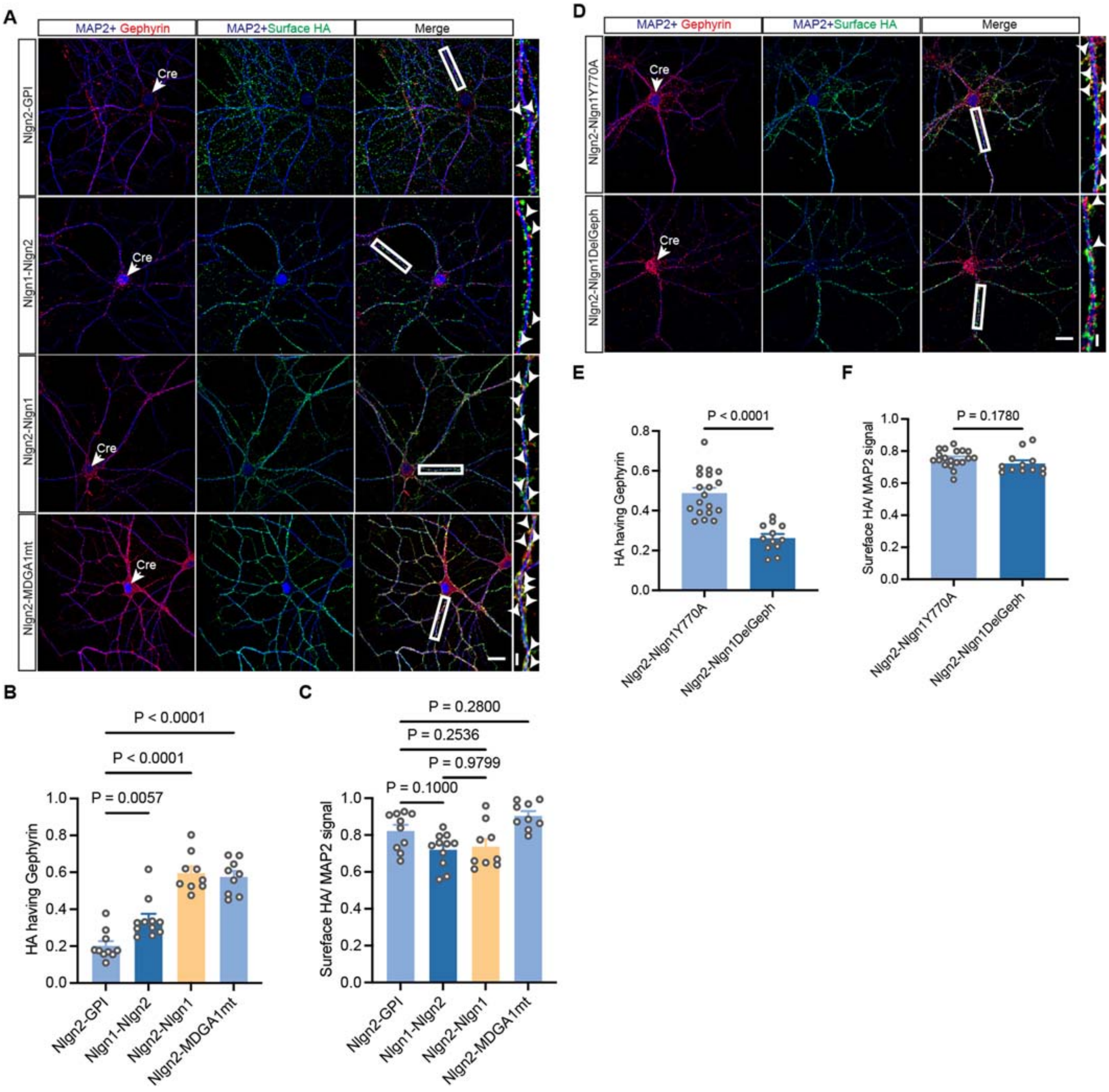


**Figure EV2. Nlgn2 is specifically localized on inhibitory synapses and is determined by the extracellular sequence of Nlgn2 and cytoplasmic gephyrin binding motif is required whereas tyrosine phosphorylation is not (related to Figs. 2, 3 and 5).**

(**A**) Representative image from DIV14-16 cultured Nlgn1234 conditional knockout mice neurons, infected with Cre (blue) and Nlgn2-GPI, Nlgn1-Nlgn2, Nlgn2-Nlgn1, and Nlgn2-MDGA1mt (from top to bottom). The neurons were labeled with antibodies to Gephyrin (red), HA (green), and MAP2 (blue). Scale bar: 20 µm. The right panels show an enlarged box area (arrowheads indicate HA puncta overlapped with Gephyrin puncta). Scale bar: 5 µm. (**B**) Summary graph of the HA-Gephyrin overlap percentage in Nlgn2-GPI, Nlgn1-Nlgn2, Nlgn2-Nlgn1, and Nlgn2-MDGA1mt conditions. (**C**) Summary graph of the surface levels of HA-tagged Nlgn2 forms relative to MAP2 signal. (**B**, **C**) (Bar and line graphs indicate mean ± SEM; numbers of cells/experiments = 10/3, 11/3, 9/3, and 9/3 for each column, left to right. Statistical significance was assessed by one-way ANOVA with post hoc Dunnett's Multiple comparisons, Nonsignificant $p > 0.05$; $^{**}p < 0.01$; $^{****}p < 0.0001$). (**D**) Representative image from DIV14-16 cultured Nlgn1234 conditional knockout mice neurons, infected with Cre (blue) and Nlgn2-Nlgn1Y770A and Nlgn2-Nlgn1DelGeph. The neurons were labeled with antibodies to Gephyrin (red), HA (green), and MAP2 (blue). Scale bar: 20 µm. The right panels show an enlarged box area (arrowheads indicate HA puncta overlapped with Gephyrin puncta). Scale bar: 5 µm. (**E**) Summary graph of the HA-Gephyrin overlap percentage in Nlgn2-Nlgn1Y770A and Nlgn2-Nlgn1DelGeph conditions. (**F**) Summary graph of the surface levels of HA-tagged Nlgn2-Nlgn1Y770A and Nlgn2-Nlgn1DelGeph relative to MAP2 signal. (**E**, **F**) (Bar and line graphs indicate mean ± SEM; numbers of cells/experiments = 19/3 and 12/3 for each column, left to right. Statistical significance was assessed by one-way ANOVA with post hoc Dunnett's Multiple comparisons, Nonsignificant $p > 0.05$; $^{****}p < 0.0001$). Source data are available online for this figure.

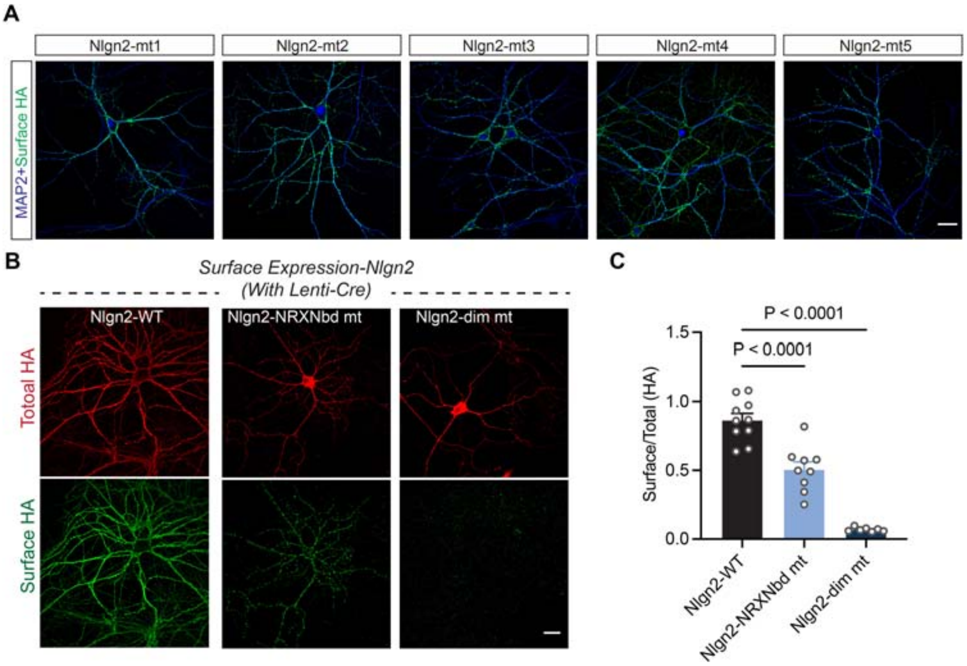


**Figure EV3. All Nlgn2 intracellular domain truncation constructs can properly traffic to the neuron membrane surface, but Nlgn2-NRXNmt and Nlgn2-dimmt constructs traffic to the neuron membrane surface signification decrease compared to the Nlgn2-WT construct (related to Fig. 4).**

(**A**) Representative image from DIV14-16 cultured Nlgn1234 conditional knockout mice neurons, infected with Cre (blue) and Nlgn2-mt1, Nlgn2-mt2, Nlgn2-mt3, Nlgn2-mt4 and Nlgn2-mt5 (from left to right). The neurons were labeled with antibodies to HA (green) and MAP2 (blue). Scale bar: 20 μm. (**B**) Representative image from DIV14-16 cultured Nlgn1234 conditional knockout mice neurons, infected with Nlgn2-WT, Nlgn2-NRXNmt, and Nlgn2-dimmt. The neurons were labeled with antibodies to total HA (red) and surface HA (green). Scale bar: 20 μm. (**C**) Summary graph of the surface HA/total HA in Nlgn2-WT, Nlgn2-NRXNmt, and Nlgn2-dimmt conditions (Bar and line graphs indicate mean ± SEM. numbers of cells/experiments = 10/3, 9/3 and 7/3 for each column, left to right. Statistical significance was assessed by one-way ANOVA with post hoc Dunnett's Multiple comparisons, ****$p < 0.0001$). Source data are available online for this figure.

 © The Author(s)

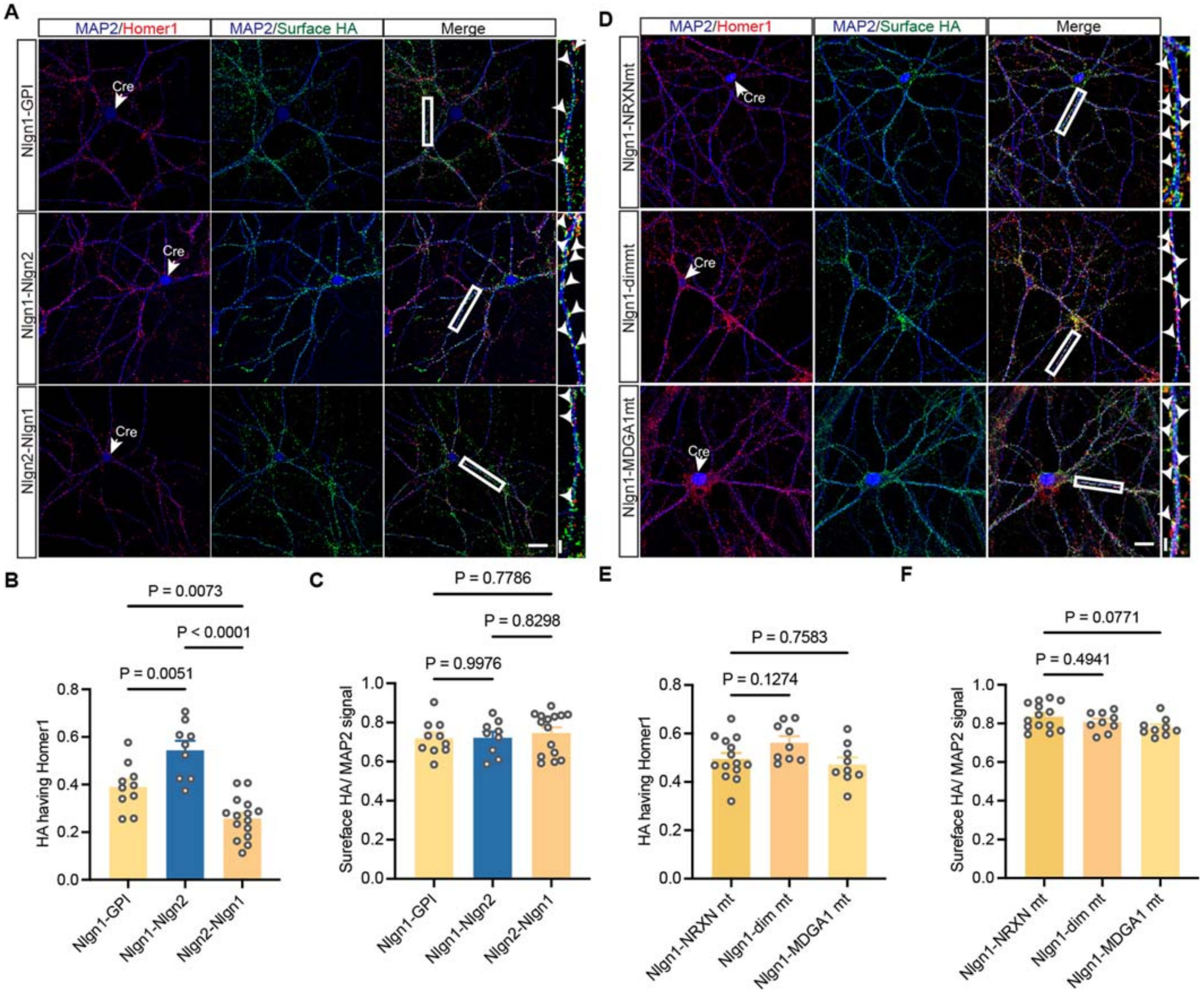


**Figure EV4. Nlgn1 is specifically localized on excitatory synapses is determined by the extracellular sequence of Nlgn1, and does not require Neurexin-binding, MDGA-binding, or dimerization (related to Figs. 6 and 7).**

(**A**) Representative image from DIV14-16 cultured Nlgn1234 conditional knockout mice neurons, infected with Cre (blue) and Nlgn1-GPI, Nlgn1-Nlgn2, and Nlgn2-Nlgn1. The neurons were labeled with antibodies to Homer1 (red), HA (green), and MAP2 (blue). Scale bar: 20 µm. The right panels show an enlarged box area (arrowheads indicate HA puncta overlapped with Homer1 puncta). Scale bar: 5 µm. (**B**) Summary graph of the HA-Homer1 overlap percentage in Nlgn1-GPI, Nlgn1-Nlgn2, and Nlgn2-Nlgn1 conditions. (**C**) Summary graph of the surface levels of HA-tagged Nlgn1 forms relative to MAP2 signal. (**B**, **C**) (Bar and line graphs indicate mean ± SEM; numbers of cells/experiments = 10/3, 9/3, and 15/3 for each column, left to right. Statistical significance was assessed by one-way ANOVA with post hoc Dunnett's Multiple comparisons, Nonsignificant $p > 0.05$; $^{**}p < 0.01$; $^{****}p < 0.0001$). (**D**) Representative image from DIV14-16 cultured Nlgn1234 conditional knockout mice neurons, infected with Cre (blue) and Nlgn1-NRNXmt, Nlgn1-dimmt, and Nlgn1-MDGA1mt. The neurons were labeled with antibodies to Homer1 (red), HA (green), and MAP2 (blue). Scale bar: 20 µm. The right panels show an enlarged box area (arrowheads indicate HA puncta overlapped with Homer1 puncta). Scale bar: 5 µm. (**E**) Summary graph of the HA-Homer1 overlap percentage in Nlgn1-NRNXmt, Nlgn1-dimmt, and Nlgn1-MDGA1mt conditions. (**F**) Summary graph of the surface levels of HA-tagged Nlgn1 forms relative to MAP2 signal. (**E**, **F**) (Bar and line graphs indicate mean ± SEM; numbers of cells/experiments = 14/3, 9/3, and 9/3 for each column, left to right. Statistical significance was assessed by one-way ANOVA with post hoc Dunnett's Multiple comparisons, Nonsignificant $p > 0.05$). Source data are available online for this figure.

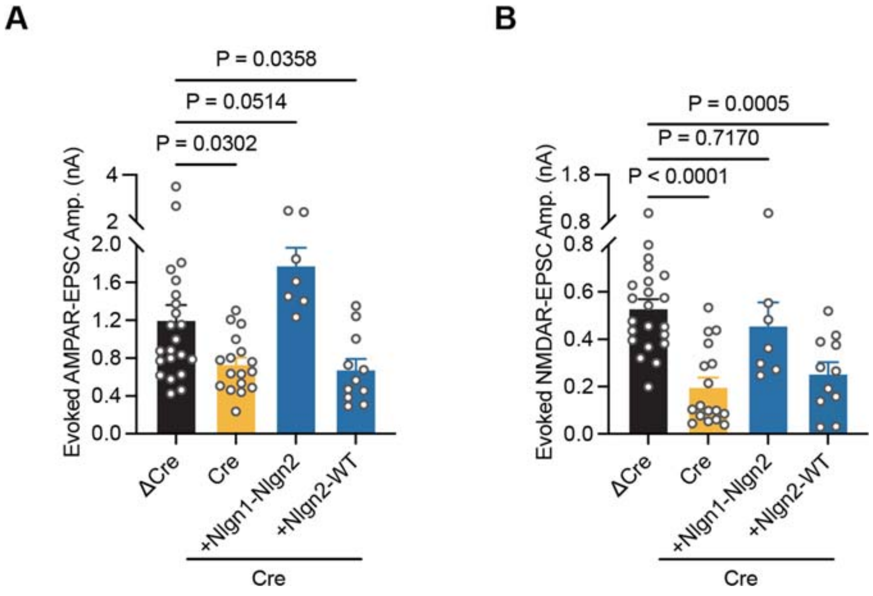


**Figure EV5. Nlgn1-Nlgn2 is sufficient for the glutamatergic synaptic transmission function of Nlgn1, but Nlgn2-WT doesn't (related to Fig. 6).**

(**A**) Summary graph of evoked AMPAR-EPSC amplitude in all conditions (Bar and line graphs indicate mean ± SEM; numbers of cells/experiments = 22/4, 17/4, 22/4, 7/2, and 11/3 for each column, left to right). Nonsignificant $p > 0.05$; *$p < 0.05$, one-way ANOVA with post hoc Dunnett's Multiple comparisons. Nonsignificant relations are indicated as ns. Note that we rescued Nlgn1-Nlgn2 and Nlgn2-WT here from the same batches of Fig. 6, so the ΔCre and Cre data here are the same as in Fig. 6. (**B**) Summary graph of evoked NMDAR-EPSC amplitude in all conditions (Bar and line graphs indicate mean ± SEM; numbers of cells/experiments = 22/4, 17/4, 22/4, 7/2, and 11/3 for each column, left to right). Nonsignificant $p > 0.05$; ***$p < 0.001$; ****$p < 0.0001$, one-way ANOVA with post hoc Dunnett's Multiple comparisons. Nonsignificant relations are indicated as ns. Note that we rescued Nlgn1-Nlgn2 and Nlgn2-WT here from the same batches of Fig. 6, so the ΔCre and Cre data here are the same as in Fig. 6. Source data are available online for this figure.

 © The Author(s)
